# Beta HPV38 oncoproteins act with a hit-and-run mechanism in ultraviolet radiation-induced skin carcinogenesis in mice

**DOI:** 10.1371/journal.ppat.1006783

**Published:** 2018-01-11

**Authors:** Daniele Viarisio, Karin Müller-Decker, Rosita Accardi, Alexis Robitaille, Matthias Dürst, Katrin Beer, Lars Jansen, Christa Flechtenmacher, Matthias Bozza, Richard Harbottle, Catherine Voegele, Maude Ardin, Jiri Zavadil, Sandra Caldeira, Lutz Gissmann, Massimo Tommasino

**Affiliations:** 1 Deutsches Krebsforschungszentrum, Heidelberg, Germany; 2 International Agency for Research on Cancer, World Health Organization, Lyon, France; 3 Department of Gynecology, Jena University Hospital - Friedrich Schiller University, Jena, Germany; 4 Department of Pathology, University Hospital of Heidelberg, Heidelberg, Germany; 5 Department of Botany and Microbiology (honorary member), King Saud University, Riyadh, Saudi Arabia; University of Wisconsin Madison School of Medicine and Public Health, UNITED STATES

## Abstract

Cutaneous beta human papillomavirus (HPV) types are suspected to be involved, together with ultraviolet (UV) radiation, in the development of non-melanoma skin cancer (NMSC). Studies in *in vitro* and *in vivo* experimental models have highlighted the transforming properties of beta HPV E6 and E7 oncoproteins. However, epidemiological findings indicate that beta HPV types may be required only at an initial stage of carcinogenesis, and may become dispensable after full establishment of NMSC. Here, we further investigate the potential role of beta HPVs in NMSC using a Cre-loxP-based transgenic (Tg) mouse model that expresses beta HPV38 E6 and E7 oncogenes in the basal layer of the skin epidermis and is highly susceptible to UV-induced carcinogenesis. Using whole-exome sequencing, we show that, in contrast to WT animals, when exposed to chronic UV irradiation K14 HPV38 E6/E7 Tg mice accumulate a large number of UV-induced DNA mutations, which increase proportionally with the severity of the skin lesions. The mutation pattern detected in the Tg skin lesions closely resembles that detected in human NMSC, with the highest mutation rate in p53 and Notch genes. Using the Cre-lox recombination system, we observed that deletion of the viral oncogenes after development of UV-induced skin lesions did not affect the tumour growth. Together, these findings support the concept that beta HPV types act only at an initial stage of carcinogenesis, by potentiating the deleterious effects of UV radiation.

## Author summary

Many epidemiological and biological findings support the hypothesis that beta HPV types cooperate with UV radiation in the induction of NMSC, the most common form of human cancer. We have previously shown that K14 HPV38 E6/E7 Tg mice, when exposed to long-term UV radiation, developed NMSC, whereas WT animals subjected to identical treatments did not develop any type of skin lesions. Here, we show that the high skin cancer susceptibility of these Tg animals tightly correlates with their tendency to accumulate UV-induced mutations in genes that are frequently mutated in human NMSC. Importantly, deletion of the HPV38 E6 and E7 genes in existing skin lesions did not affect the further growth of the cancer cells. Together, these findings support the model that beta HPV infection is a co-factor in skin carcinogenesis, facilitating the accumulation of the UV-induced DNA mutations.

## Introduction

Non-melanoma skin cancer (NMSC) is the most common cancer in adult Caucasian populations [1]. The cutaneous human papillomavirus (HPV) types belonging to genus beta are suspected, together with ultraviolet (UV) radiation, to be involved in NMSC [2,3]. The first two beta HPV types, 5 and 8, were isolated from skin lesions of patients with a disorder called epidermodysplasia verruciformis (EV). EV patients are highly susceptible to beta HPV infection in the skin and develop cutaneous squamous cell carcinoma (cSCC) at anatomical sites exposed to sunlight [4]. The fact that organ transplant recipients, due to their immunosuppressed status, have an elevated risk of beta HPV infection and development of cSSC provided evidence for the role of beta HPV types in skin carcinogenesis also in non-EV individuals [5,6]. Finally, many epidemiological studies support the link between these viruses and cSCC in the general population [2,3,7]. These studies showed that, compared with the general population, patients with a history of cSCC are more frequently positive for viral DNA in the skin and/or for antibodies against the major capsid protein L1.

Molecular analysis showed that not all cancer cells contain a copy of the beta HPV genome and that the copy number of the beta HPV genome is higher in pre-malignant actinic keratosis (AK), a precursor lesion of SCC, than in SCC [8]. Thus, these data suggest that beta HPV types may act at an initial stage of skin carcinogenesis and that after full transformation of the infected cells, viral DNA can be lost. This model is consistent with the fact that additional carcinogens are involved in skin carcinogenesis. Considering that UV radiation is the key risk factor for cSSC development [9–11], the most plausible hypothesis is that beta HPV types exacerbate the accumulation of a large number of UV-induced somatic mutations, facilitating cellular transformation. Subsequently, the expression of the viral oncogenes may become irrelevant for the maintenance of the malignant phenotype.

Several studies in human keratinocytes, the natural host of beta HPV types, showed that E6 and E7 from some beta HPV types target key pathways linked to DNA repair, apoptosis, and cellular transformation [3]. Several transgenic (Tg) models for beta HPV have been generated [12–16], some of which have highlighted the synergism between viral oncogene expression in the skin epithelium and UV radiation in promoting cSCC [3]. Tg mice expressing beta HPV38 E6 and E7 in the basal layer of the epidermis under the control of the cytokeratin K14 promoter (K14) did not spontaneously develop any lesions during their life span. Upon long-term exposure to UV radiation (30 weeks), they developed first skin lesions closely resembling human AK and subsequently cSCCs. In contrast, wild-type (WT) mice developed neither pre-malignant lesions nor cSCCs when exposed to the same dose of UV radiation [15]. However, it is still unknown whether the high susceptibility of the K14 HPV38 E6/E7 Tg animals to UV-induced skin carcinogenesis is linked to the accumulation of mutations facilitated by the viral oncoproteins, which may become dispensable after cSCC development. In this study, we addressed this open question on the synergism between UV radiation and beta HPV38 E6 and E7 oncoproteins using the Tg mouse model. We showed that viral oncoproteins act at an initial stage of UV-induced skin carcinogenesis, facilitating the accumulation of a large number of somatic mutations in crucial genes that are associated with cSCC development in humans. In addition, silencing of the expression of the viral genes in established skin lesions does not affect further tumour growth.

## Results

### Expression of HPV38 E6 and E7 in mouse skin facilitates the accumulation of UV-induced DNA mutations

We have previously shown that HPV38 E6/E7 expression in mouse skin strongly increases susceptibility to UV-induced carcinogenesis [15]. To evaluate whether the development of skin lesions present in K14 HPV38 E6/E7 Tg mice of chronic UV irradiation correlated with the number of accumulated DNA mutations, we used whole-exome sequencing of WT and Tg samples.

For this analysis, we selected normal skin from WT mice not exposed or exposed to UV radiation for 30 weeks (*n* = 2) and histologically confirmed skin specimens from three independent K14 HPV38 E6/E7 Tg mice UV-irradiated for 30 weeks, i.e., (i) normal skin, (ii) pre-malignant skin lesions and (iii) cSCC. For the pre-malignant lesions, the histological analyses revealed that they have the classic features observed in humans of the precancerous condition of AK, including slight atypia, parakeratosis, and acanthosis (S1 Fig) [15]. Exome sequencing (Illumina Hi-Seq) of collected samples generated an average coverage of 141.71× ± 11.9 (mean ± standard deviation).

The genomic sequence of the WT mouse not exposed to UV radiation was used as a control sample in paired analysis. Only 10 mutations were detected in the skin of the UV-irradiated WT mouse. Similarly, less than 10 mutations were detected in the Tg mouse not exposed to UV irradiation. In both cases, all the mutations were in genes not directly linked to carcinogenesis (S1 Table).

**Fig 1:**
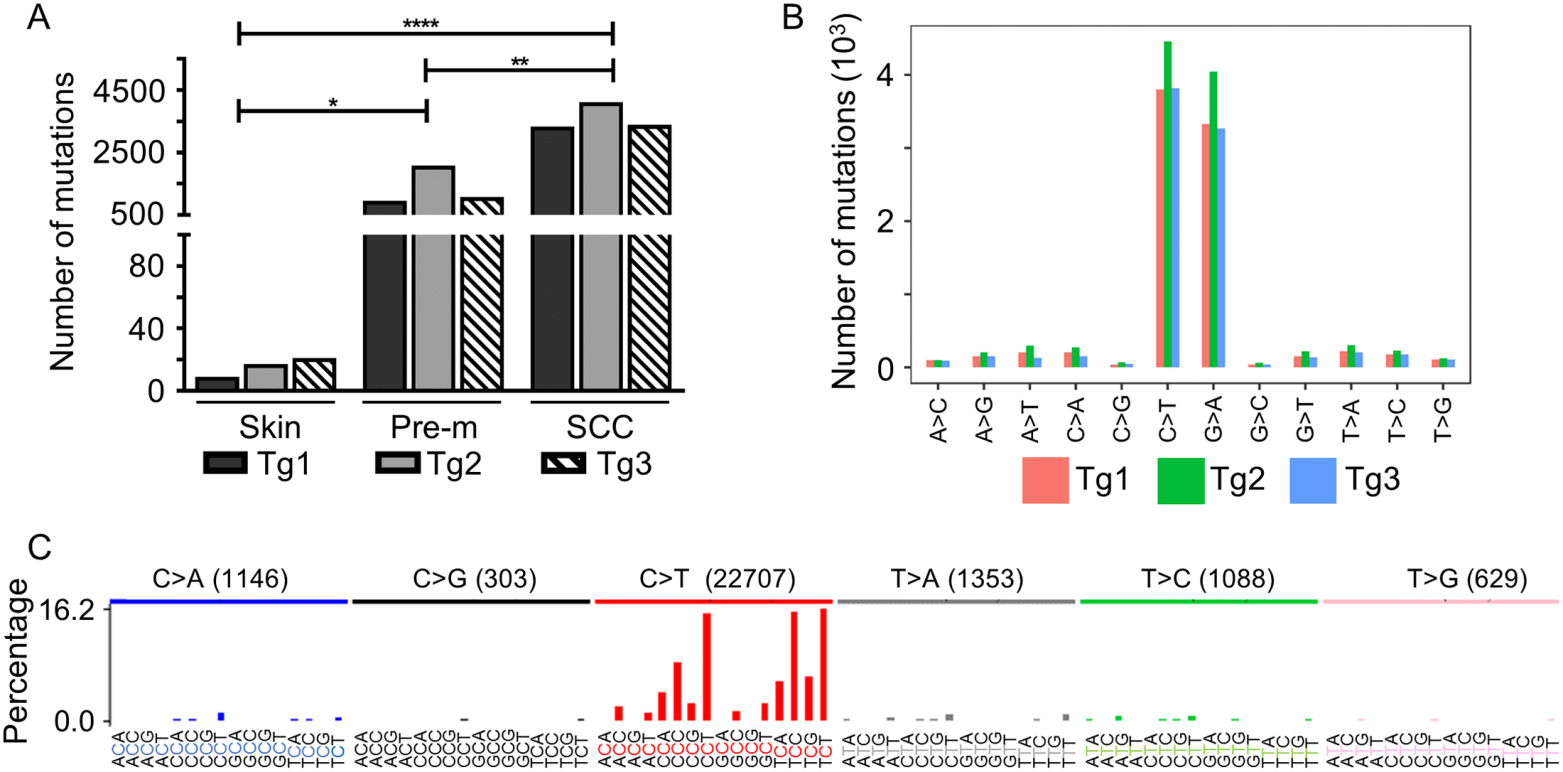
HPV38 E6 and E7 induce an increased steady-state level of UV-induced mutations in mouse skin keratinocytes. (A) UV-induced cSCCs in K14 HPV38 E6/E7 Tg mice have a vast number of somatic mutations. SCCs display a very high mutational load, with each Tg animal (Tg1–3) harbouring almost 3 times the number of variants compared with pre-malignant lesions (Pre-m). All differences in number of DNA mutations among the tree types of specimens were statistically significant: * ≤0.05; ** ≤0.01; **** ≤0.0001. (B) cSCCs of K14 HPV38 E6/E7 Tg mice display the classic UV-induced mutation signature with a very high number of C:G > T:A mutations. This type of mutation represents the majority of the SNV type in SCC samples of the three Tg animals. (C) Mutation spectrum of pooled SCC samples from the three mice. This spectrum displays the high prevalence of C:G > T:A mutations, especially in the 5′-T_N-3′ and 5′-C_N-3′ context. The *y* axis represents the percentage of mutations, and the *x* axis the trinucleotide sequence context.

In UV-irradiated Tg animals, the mutational load varied across our cohort of well-differentiated cSCC exomes, averaging 3541 somatic variants (range, 3261–4027) or 68.58 ± 7.64 variants per Mb. The exome of the pre-malignant samples had substantially fewer variants, with an average of 1337 somatic variants (range, 937–2026) or 23.14 ± 14.70 variants per Mb. The exome of the chronically UV-exposed normal skin of Tg mice harboured an average of 15 somatic variants (range, 11–20) or 0.29 ± 0.08 variants per Mb (S2 Table). Thus, the number of somatic mutations was proportional to the severity of the skin lesion; the average number in SCCs was approximately double that in the pre-malignant lesions (Fig 1A).

The vast majority of the somatic mutations detected in SCCs were C:G > T:A mutations, mutations that are also prevalent in the UV-induced mutational signature (Fig 1B and 1C). We applied the non-negative matrix factorization (NMF) method to extract the mutational signatures composed of 96 single base substitution (SBS) types considering the sequence context (one base upstream and one base downstream) (S2 Fig). The extracted signature was compared with known mutational signatures by the cosine similarity method [17,18]. The value of the similarity obtained for the new B signature is 0.86 for COSMIC signature 27 (UV signature) (S2 Fig), indicating the clear prevalence of the impact of UV radiation on the etiology of these cSCCs.

**Fig 2:**
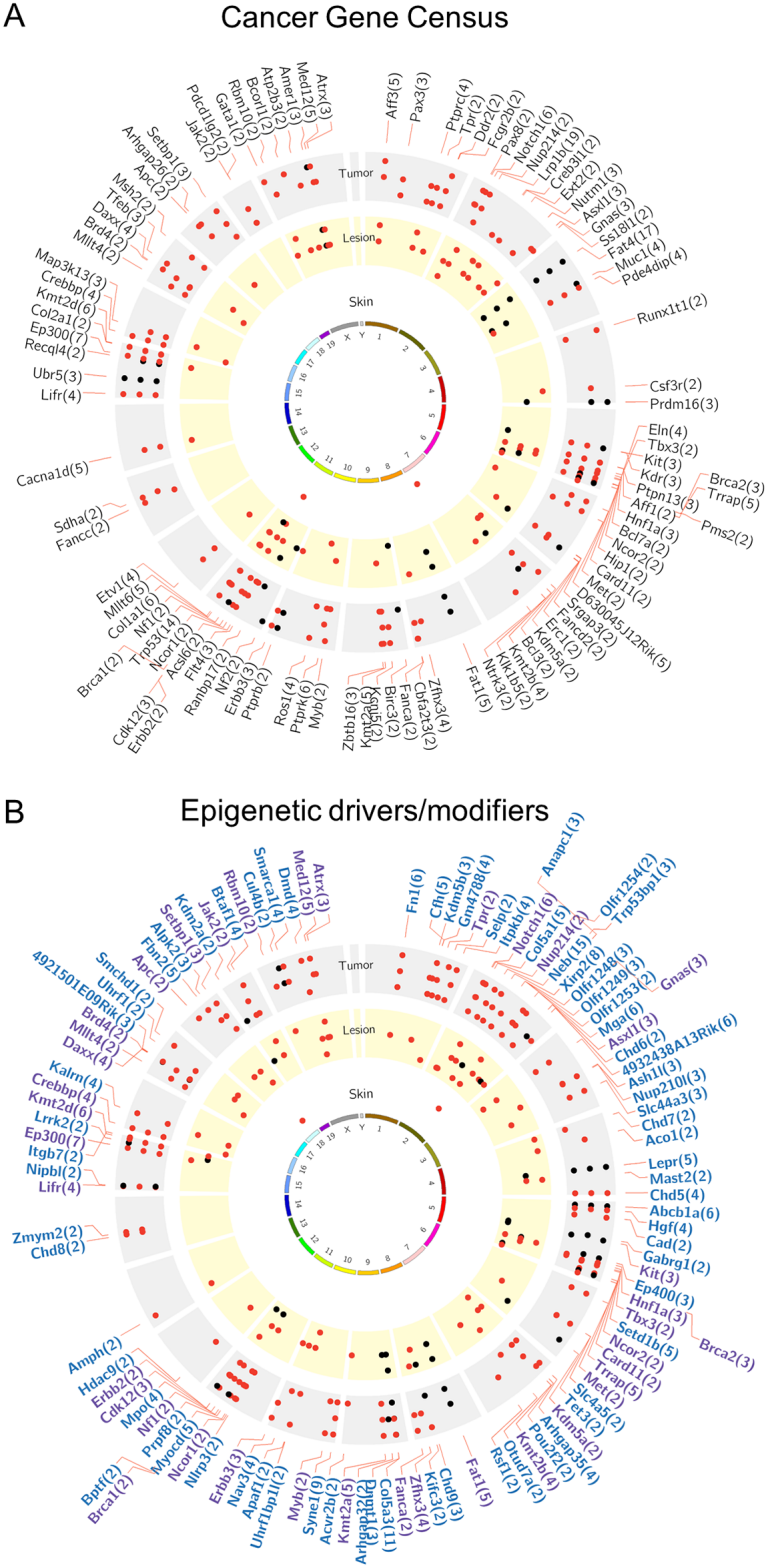
Cancer-related genes recurrently mutated in cSCCs of K14 HPV38 E6/E7 Tg mice. (A) Circos presentation of mutations occurring in the same genes between the different mice. From the centre to the outside, the skin samples (white), the lesion samples (yellow), and the SCC samples (grey) are displayed for *n* = 3 mice each. Each track (three per colour) corresponds to one animal. Red dots represent C:G > T:A mutations, and black dots represent the other types of mutations. For Circos A, only the mutations that occur in genes present in the Cancer Gene Census list from the COSMIC database are displayed, with the number of recurrent mutations in these genes in parentheses. (B) For the epigenetic drivers/modifiers, only the mutations that occur in the epi-driver or the epi-modifier gene lists are displayed. Blue gene names correspond to genes that are only involved in epigenetic processes, and purple gene names correspond to genes that are involved in epigenetic processes and that are present in the Cancer Gene Census list. The total number of recurrent mutations occurring in each of these genes is also displayed in parentheses.

To assess the biological significance of the somatic mutations detected in the skin lesions of the K14 HPV38 E6/E7 Tg mice, we determined whether they were detected in the previously compiled lists of epi-driver and epi-modifier genes [19–23], as well as genes identified in the Cancer Gene Census [24]. As shown in Fig 2, three classes of genes were found to be recurrently mutated in pre-malignant and malignant skin lesions of K14 HPV38 E6/E7 Tg animals, suggesting a selective process for the enrichment of mutations in these groups of genes.

Pathway analyses confirmed that the mutations detected in mouse cSCC affect key pathways intimately linked to cellular transformation (S3 Table).

**Fig 3:**
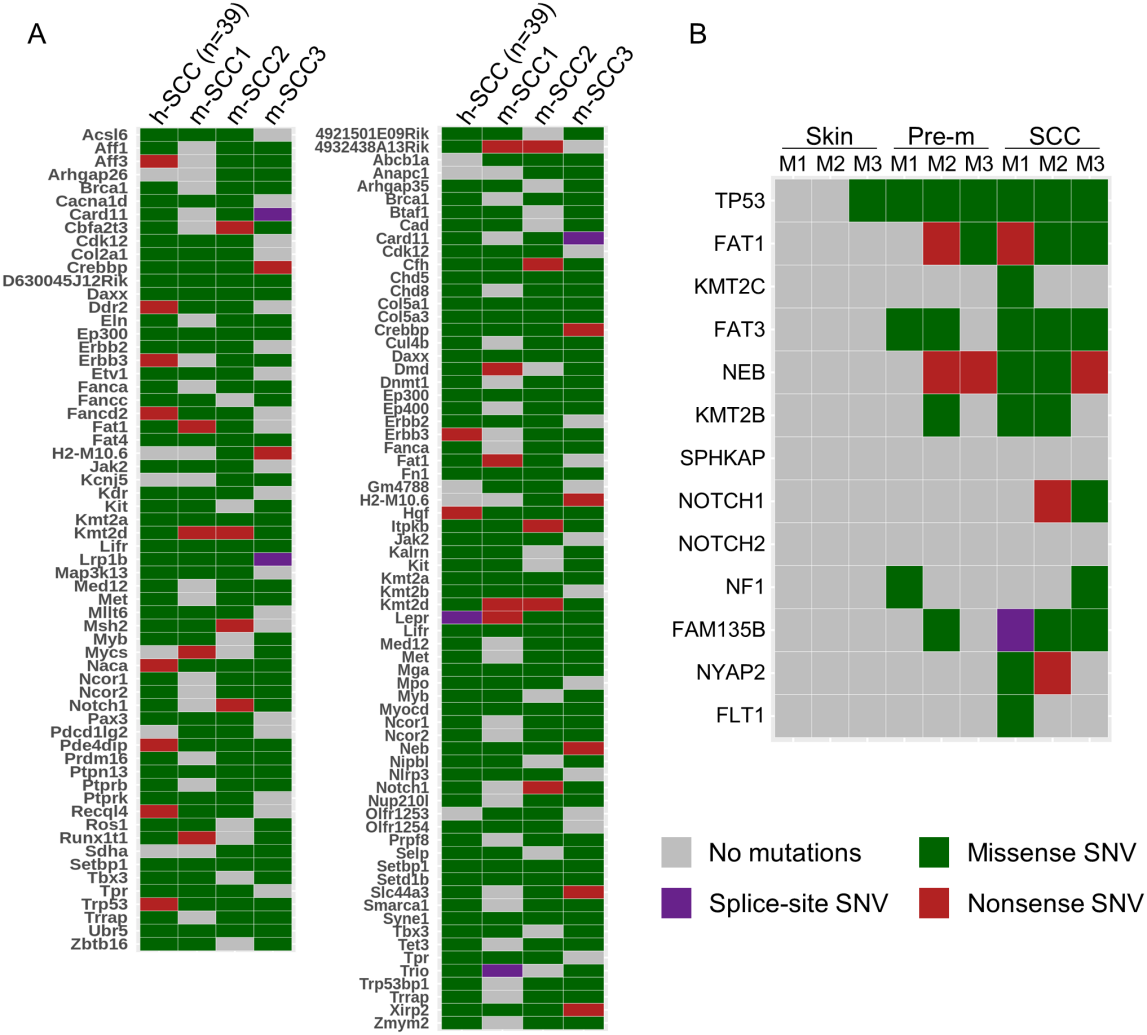
Several genes mutated in human skin lesions are also mutated in the UV-induced skin lesions of cSCCs of K14 HPV38 E6/E7 Tg mice. (A) Heatmap of significantly mutated genes, corresponding to genes recurrently mutated in at least two mouse SCC samples and reported in the Cancer Gene Census list from the COSMIC database (left panel) or having an impact on epigenetic regulation processes (right panel). The types of mutation represented by colours are chosen according to the most prevalent mutation type in each sample. The data for the human samples displayed in the first column are derived from a previous publication on cutaneous SCC. (B) Heatmap of mutations in genes in normal skin, pre-malignant lesions, and cSCC from different mice (M1–3) reported as significantly mutated in human cSCC.

A comparison of somatic mutations detected in our experimental Tg mouse model and in human cSCC [25] revealed that a large number of epi-driver, epi-modifier, and Cancer Gene Census genes were recurrently mutated in murine and human cSCC (Fig 3A).

A recent study identified the top human genes mutated in cSCC [26]. Interestingly, most of these genes are also found to be mutated in the UV-induced skin lesions of the K14 HPV38 E6/E7 Tg animals (Fig 3B). In agreement with previous findings on human cSCC [25], Trp53 showed up as the most mutated gene in the murine Tg-derived cSCC (Figs 2A and 3B). Here, p53 mutations appear to be an early event in skin carcinogenesis, because they were detected in one sample of normal skin as well as in all pre-malignant lesions and cSCCs. In agreement with our data, it was reported that p53 mutations can be detected in keratinocytes of UV-exposed normal skin [27,28]. However, all mutations were identified in the p53 DNA-binding domain (S4 Table), supporting their key role in the process of carcinogenesis. Consistent with the fact that in keratinocytes the Notch signalling pathway promotes cell-cycle exit and differentiation [29,30], *NOTCH1* and *NOTCH2* have been found to be mutated in human cSCC [25]. In our Tg mouse model, mutated *NOTCH1* and/or *NOTCH2* were also detected in all three cSCCs, but never in pre-malignant lesions (Fig 3B).

**Fig 4:**
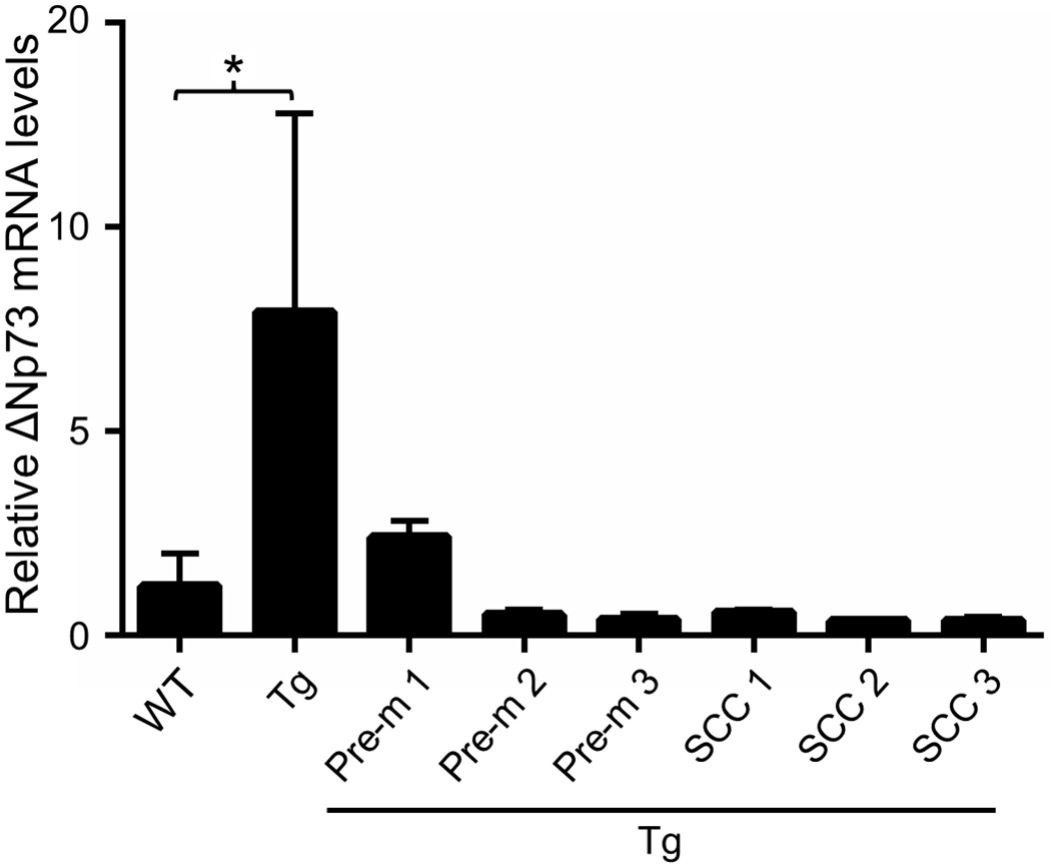
ΔNp73α mRNA levels are high in the skin of HPV38 E6/E7 Tg mice, but are decreased in the UV-induced skin lesions harbouring p53 mutations. Total RNA was extracted from the skin of WT (*n* = 4) or K14 HPV38 E6/E7 Tg animals (*n* = 5) as well as histologically confirmed pre-malignant (pre-m) and SCC from three independent mice and harbouring mutated p53. ΔN73α levels were measured by quantitative RT-PCR. The data shown are the mean of two independent experiments. The differences in ΔN73α mRNA levels between WT and K14 HPV38 E6/E7 Tg animals were statistically significant: * <0.05.

Our previous data showed that HPV 38 E6 and E7 expression in human keratinocytes resulted in accumulation of TAp53, which is recruited to the internal promoter located in intron 3 of p53 gene, with resulting transcriptional activation of ΔNp73α [31,32]. Fig 4 shows that also in the mouse skin, expression of the viral genes leads to increased ΔNp73α transcription. In contrast, in histologically confirmed pre-malignant and SCC lesions, p53 mutation correlates with a strong decrease in ΔNp73α mRNA levels (Fig 4).

In conclusion, our findings show that the expression of HPV38 E6 and E7 oncogenes in mouse skin increases susceptibility to UV-induced cSCC by facilitating the accumulation of somatic mutations that have been clearly associated with skin cancer development in humans.

### HPV38 E6 and E7 play a role at initial stages of UV-induced skin carcinogenesis but are not required for cancer maintenance

Many studies support the role of beta HPV types, together with UV radiation, in the development of skin SCC [2,3]. However, in contrast to the mucosal high-risk HPV types such HPV16 that are required in all steps of cervical carcinogenesis, beta HPV types appear to have a role in the initial steps of carcinogenesis. To test this hypothesis, we constructed our K14 HPV38 E6/E7 Tg mice as a conditional expression model with two loxP elements, located immediately upstream and downstream of the viral genes [15]. Originally, we crossed the K14 HPV38 E6/E7 Tg mice with K14 Cre-ERT2 Tg animals overexpressing the Cre recombinase gene fused to a triple-mutant form of the human estrogen receptor that gains access to the nuclear compartment only after exposure to 4-hydroxytamoxifen (TMX) but not to the natural ligand 17β-estradiol, in order to silence E6/E7 expression by Cre-mediated deletion of the floxed viral genes at different times of the chronic UV irradiation, i.e., different stages of SCC development. Although the expression of the viral genes could be efficiently silenced upon administration of TMX to 5-week-old K14 Cre-ERT2 HPV38 E6/E7 compound mice, in the compound mice a strong decrease in viral gene expression was observed during the 30 weeks of UV irradiation in the absence of TXM treatment (S3 Fig). The loss of HPV38 E6 and E7 genes in long-term experiments was most likely due to a basal, non-specific Cre recombinase activity in the nucleus of mouse skin keratinocytes. None of the K14 Cre-ERT2 HPV38 E6/E7 Tg compound lines developed cSCC after 30 weeks of UV irradiation, further highlighting the importance of the viral proteins in UV-induced carcinogenesis.

**Fig 5:**
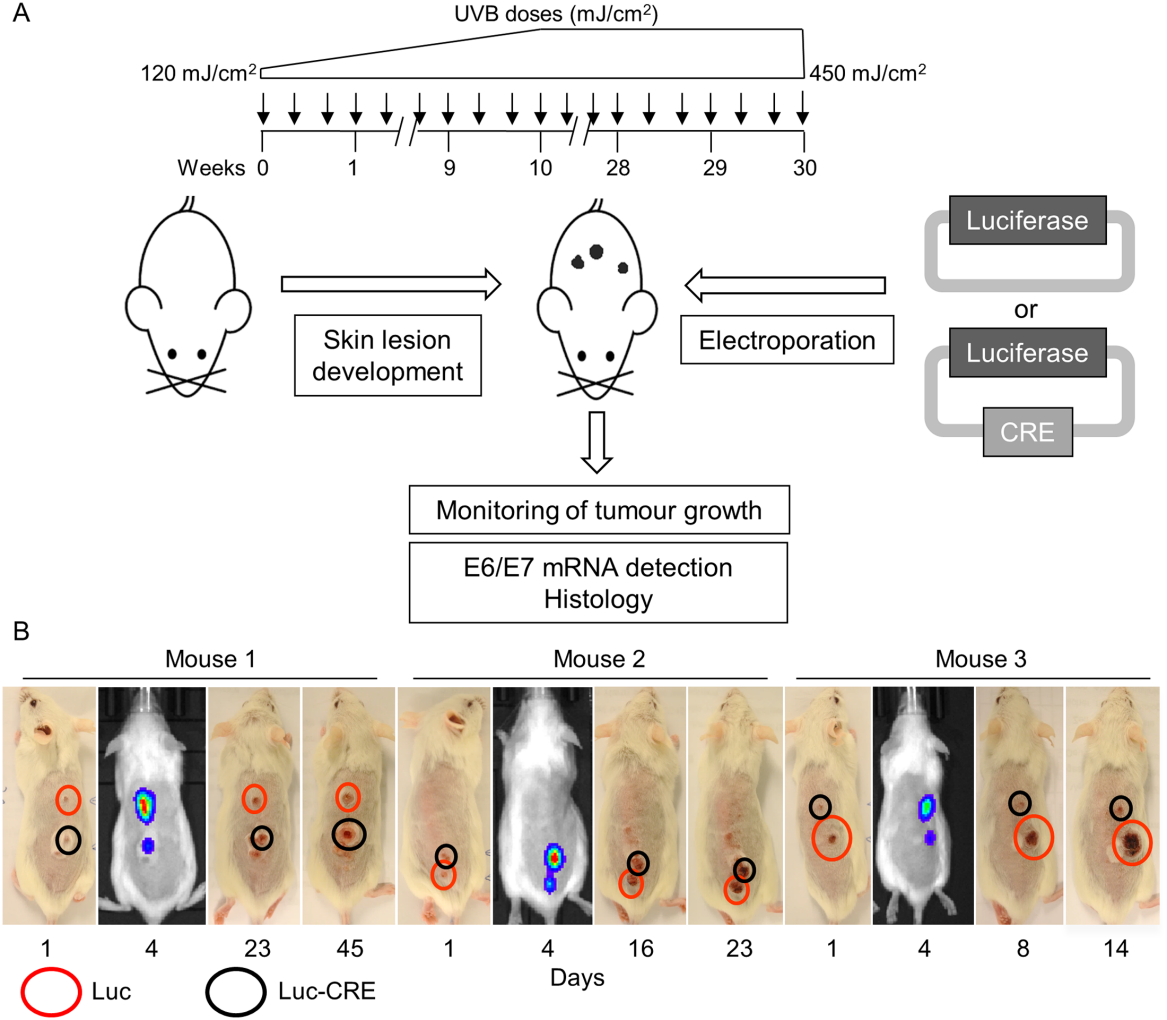
Luciferase expression vectors can be efficiently electroporated into skin lesions of K14 HPV38 E6/E7 Tg mice. (A) Schematic diagram of the electroporation procedure of skin lesions of K14 HPV38 E6/E7 Tg mice. (B) Luciferase activity is detected in lesions electroporated with the control vector (Luc) as well as in lesions electroporated with the plasmid coding for the Cre recombinase and luciferase genes (CRE-Luc). Mice and tumour growth are closely monitored at regular intervals.

Therefore, we developed a different strategy to evaluate the requirement of HPV38 E6 and E7 genes for cancer maintenance (Fig 5A). K14 HPV38 E6/E7 Tg mice were exposed to long-term UV irradiation, and after the appearance of well-defined skin lesions, after about 22–25 weeks of irradiation, two different DNA vectors were delivered by electroporation into the abnormal tissues. Because of the small size of the electroporated skin lesions, we could not perform any biopsy; therefore, we did not have any histological information about whether they correspond to pre-malignant or malignant lesions. Results obtained in several independent experiments showed that the lesions that occurred after 22–25 weeks of UV irradiation correspond to pre-malignant lesions or an early stage of cSCC [15,16]. Both vectors contain a scaffold/matrix attachment region (S/MAR) that keeps the plasmid in an episomal state, avoiding any integration-mediated toxicity, and ensures robust and persistent gene expression [33]. The vector codes for luciferase and Cre recombinase genes (Cre-Luc) separated by the P2A cleavage site, whereas the control vector expresses only a luciferase gene (Luc). Luciferase was used to monitor the efficiency of transfection by non-invasive *in vivo* imaging, and Cre was used to induce the excision of the viral genes. A total of 23 lesions on 14 mice were transfected either with the Luc vector (*n* = 9) or with the Cre-Luc vector (*n* = 14). When possible, the same mouse was injected with both vectors, each on a different lesion. Three representative mice are shown in Fig 5B. Luciferase activity was detected in the animals’ skin in each of the electroporated areas independently of the vector type.

**Fig 6:**
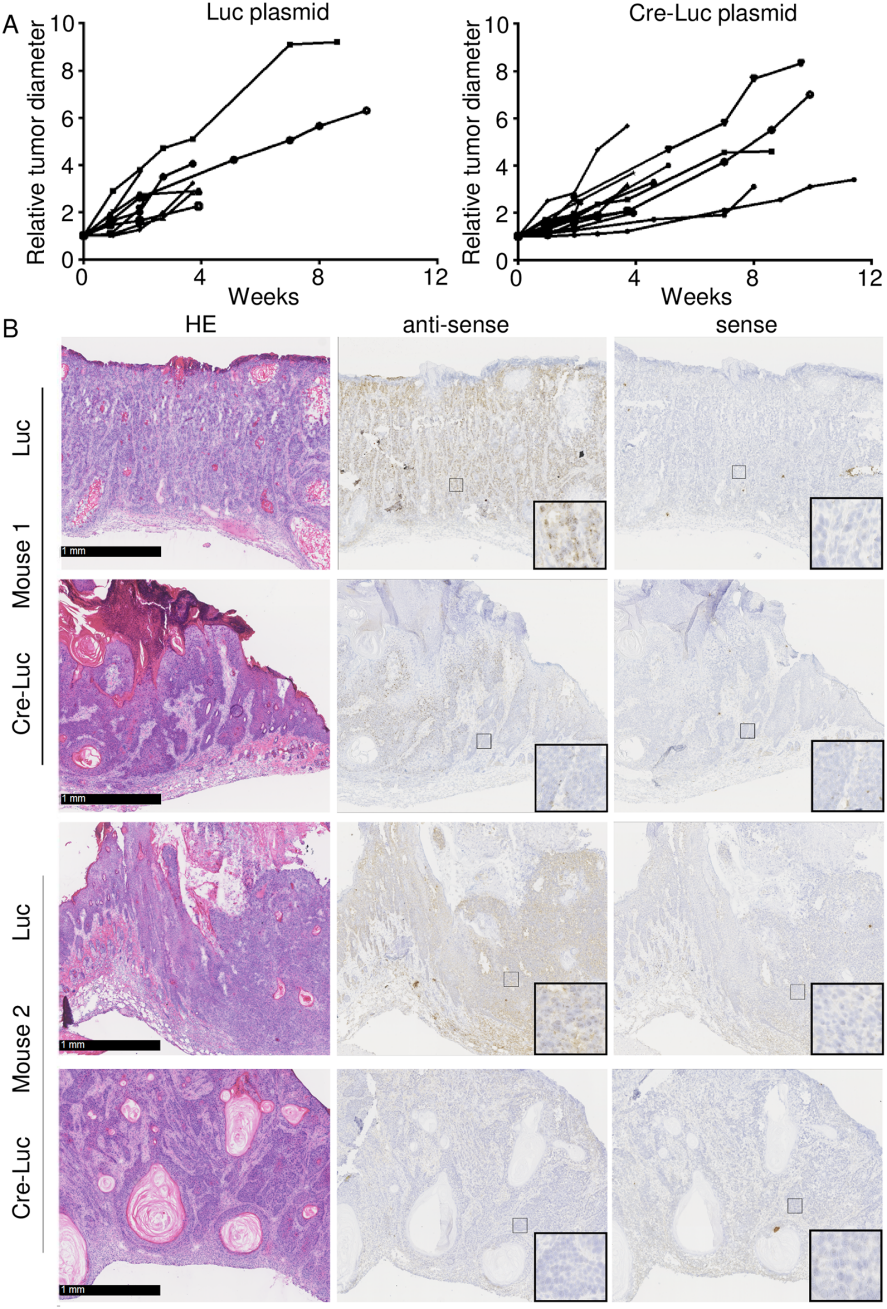
HPV38 E6 and E7 expression is not required for the viability of cancer cells in K14 HPV38 E6/E7 Tg mice. (A) Electroporated lesions were kept under control and the diameter was recorded weekly. On the day of injection, the lesion diameter varied between 1.2 mm and 2.5 mm for the lesions injected with the Luc plasmid, and between 1.3 mm and 2.6 mm for the lesions injected with the Cre-Luc plasmid. To standardize the measurement, each lesion diameter was set to an arbitrary value of 1 on the day of injection, and the following measurements were adjusted accordingly. The difference in tumour growth between the lesions injected with the Luc plasmid and the lesions injected with the Cre-Luc plasmid was not significant according to an unpaired two-sample Student’s *t*-test (*p* = 0.3108, *t* = 1.052; *df* = 14). The test was run on data from the fourth week, because afterwards the number of living animals was substantially reduced. (B) Representative images of SCC sections from two different HPV38 E6/E7 Tg mice. Sections were taken from tumours initially electroporated with pS/MARt-Luc plasmid (Luc) or with pS/MARt-Luc-P2A-Cre plasmid (Cre-Luc). The morphological analysis revealed no substantial differences between the specimens; the tumours were all classified as invasive cSCC, with deep penetration into the dermis or into the muscular fibres, and clear and diffuse atypia. The loss of the viral mRNA in the tumours injected with the Cre-Luc plasmid was confirmed by *in situ* RNA hybridization using a complementary (antisense) riboprobe, while the staining with a sense probe confirmed the specificity of the signal.

After electroporation, the animals were irradiated until the end of the 30-week UV irradiation protocol and closely monitored for several weeks to evaluate the progression of the skin lesions. No significant difference in tumour growth was observed in animals transfected with the Luc or Cre-Luc vectors (Fig 6A). Histological analyses confirmed that 100% percent of the Luc-injected lesions and 93% of the Cre-Luc injected lesions (13 out of 14) evolved into invasive cSCC; a morphological examination revealed no major differences between the two groups of tumours (Fig 6B). Detection of the viral RNA transcripts by RNA-RNA *in situ* hybridization confirmed that electroporation of skin lesions with the Cre-Luc vector, but not with the Luc vector, resulted in the loss of E6/E7 expression in large islands of cancer tissue (Fig 6B).

In conclusion, our findings show that after the accumulation of UV-induced DNA mutations and the development of skin lesions, the expression of the HPV38 E6/E7 genes is dispensable for the maintenance of the malignant phenotype of skin cancer cells.

## Discussion

Although the HPV family includes more than 200 types, to date only the mucosal high-risk (HR) HPV types have been clearly associated with human carcinogenesis. These viruses are the etiological agents of cervical cancers as well as a subset of other genital and oropharyngeal cancers [34]. Beta HPV types have been proposed to be associated with cSCC. They were initially linked to cSCC in EV patients, but now many epidemiological and biological studies support the role of beta HPV types in skin carcinogenesis also in non-EV individuals [3].

We have previously shown in a Tg mouse model that expression of beta HPV38 E6 and E7 in the skin strongly increases the risk of cSCC development upon UV irradiation [15]. Here, we showed that the higher susceptibility of K14 HPV38 E6/E7 Tg mice to UV-induced skin carcinogenesis tightly correlates with the accumulation of a high number of mutations in the keratinocyte genome. Remarkably, exposure of WT animals to the same doses of UV radiation did not lead to accumulation of DNA mutations and development of cSCC. These data suggest that the HPV38 oncoproteins can negatively affect the DNA repair machinery and/or immune pathways that lead to the elimination of damaged cells. We have recently shown that K14 HPV38 E6/E7 Tg mice are hampered in the production of interleukin 18 (IL-18) during their exposure to UV radiation [16]. Upon UV irradiation and activation of the inflammasome, keratinocytes secrete high levels of cytokines from the IL-1 family, including IL-18, thus inducing a broad spectrum of processes, such as infiltration and activation of inflammatory leukocytes, immunosuppression, DNA repair, and apoptosis [35–38]. Thus, it is likely that the high susceptibility to UV-induced DNA mutations and skin carcinogenesis of K14 HPV38 E6/E7 Tg mice may be linked to the negative impact of HPV38 on IL-18 production.

Analysis of the mutational profile revealed that a large number of genes encoding for epi-drivers or epi-modifiers and proteins known to be associated with carcinogenesis (Cancer Gene Census) harbour missense or nonsense mutations. Most importantly, the gene mutation profile found in murine cSCC shows remarkable similarities to the mutational profile found in human cSCC. In particular, mutations in p53 appear to be an early event in murine and human skin carcinogenesis. We have previously shown that beta HPV38 E7 alters the p53/73 network by inducing accumulation of p53/p73 antagonist ΔNp73α [31,32]. In human keratinocytes expressing beta HPV38 E6 and E7, ΔNp73α forms a transcriptional inhibitory complex, which binds a subset of p53-regulated promoters, preventing their activation in the presence of cellular stress [39]. Because the major role of p53 is to safeguard genome integrity, the high cancer susceptibility of K14 HPV38 E6/E7 Tg mice along with the high numbers of accumulated UV-induced DNA mutations can be explained, at least in part, by the properties of the beta HPV oncoproteins. However, once p53, and likely other cellular genes, are irreversibly inactivated by DNA mutations induced by UV radiation, the progression and maintenance of the skin carcinogenic process could become independent of the expression of viral genes. In agreement with this view, ΔNp73α mRNA levels decrease strongly in UV-induced skin lesions of K14 HPV38 E6/E7 Tg animals after accumulation of p53 mutations. In addition, we observed that the deletion of the HPV38 E6 and E7 genes does not affect further growth of the tumour. In contrast, in K14 Cre-ERT2 HPV38 E6/E7 Tg the loss of the viral genes at early stages of the irradiation protocol prevents the development of UV-induced skin lesions, underlining the key function of HPV38 E6 and E7 in UV-mediated carcinogenesis.

**Fig 7:**
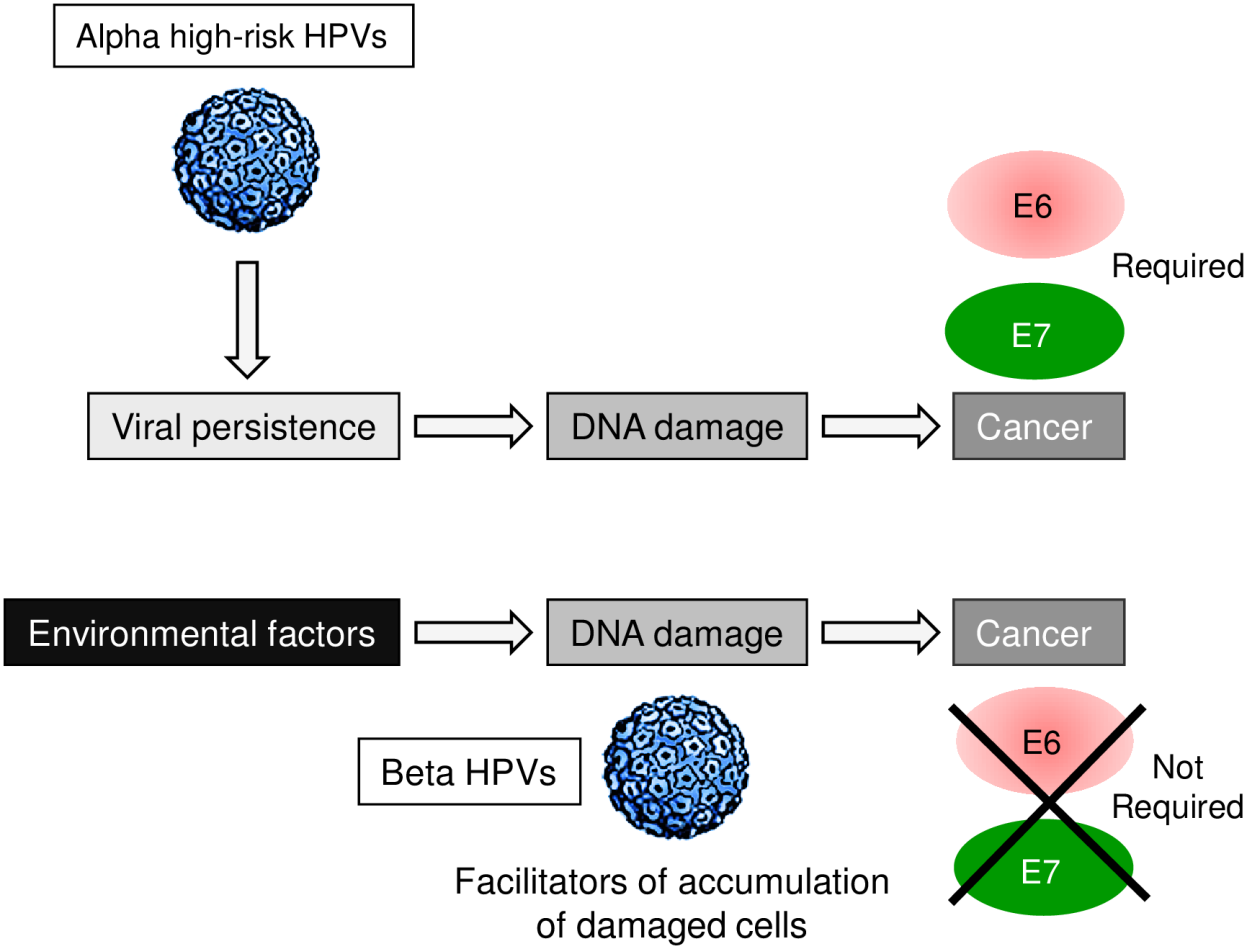
Schematic representation of well-known and hypothetical models of virus-associated carcinogenesis.

These findings in the K14 HPV38 E6/E7 Tg mouse model are in agreement with the studies on human skin lesions, supporting an early role of beta HPV types in skin carcinogenesis. Indeed, the copy numbers of the beta HPV genome appear to be higher in the pre-malignant lesion, AK, than in cSCC [8]. In addition, not all cancer cells contain a copy of a beta HPV genome [8]. Thus, the mechanisms of carcinogenesis induced by beta HPV types appear to be substantially different from those of the mucosal HR HPV types. In the case of the mucosal HR HPV types, the viral oncoproteins are the major drivers of cancer development (e.g. in the cervix) that, in addition, are required throughout the entire carcinogenic process (Fig 7). In contrast, UV-induced damage is the main carcinogen of cSCC. Here, however, beta HPV oncoproteins can facilitate the accumulation of UV-induced DNA damage but they are dispensable after full development of a malignant lesion (Fig 7).

Why do different HPV types display different biological properties? Cutaneous and mucosal HPV types infect cells at distinct anatomical sites exposed to different environmental stresses. Thus, it is not surprising that they have evolved with divergent biological properties. All HPV types rely on the DNA replication machinery of the host cell. Therefore, they must have developed several mechanisms to maintain the infected cell in a proliferative state to guarantee efficient viral genome replication. Exposure of skin keratinocytes to UV radiation leads to accumulation of DNA damage, which in turn induces cell-cycle arrest or apoptosis to allow repair or elimination, respectively, of the damaged cell. The cutaneous HPV types appear to be able to circumvent this adverse effect of UV radiation on keratinocyte proliferation, promoting the accumulation of damaged cells in the skin and, consequently, carcinogenesis.

Our previous findings showed that different HPV38 E6/E7 expression levels in independent Tg lines influence the rate of SCC development [15]. Thus, it plausible to hypothesize that also in humans, the viral gene expression levels may have an impact on UV-induced skin carcinogenesis. Limited data are available on beta E6 and E7 gene expression in normal skin and pre-malignant and malignant skin lesions (reviewed in [2,3]). There is no information on the different spliced forms of beta HPV genes and how they could determine a different efficiency in protein synthesis. Thus, additional studies are required in humans to corroborate the findings obtained in the Tg mouse model on the hit-and-run mechanism of HPV38 in UV-induced carcinogenesis.

In conclusion, our findings in a Tg mouse model highlight a novel mechanism of infection-associated carcinogenesis, in which the virus is not the driving force but synergizes with UV radiation in promoting cSCC.

## Methods

### Tg mice

The transgenic animal model FVB/NTgN(38E6E7)187DKFZ (https://mito.dkfz.de/mito/Animal%20line/10954) has been previously described [15]. UVB irradiation was performed under sevoflurane anaesthesia, and every effort was made to minimize suffering.

### Ethics statement

The animal facility of the German Cancer Research Center has been officially approved by responsible authority (Regional Council of Karlsruhe, Schlossplatz 4–6, 76131 Karlsruhe, Germany), official approval file number 35–9185.64. Housing conditions are thus in accordance with the German Animal Welfare Act (TierSchG) and EU Directive 425 2010/63/EU. Regular inspections of the facility are conducted by the Veterinary Authority of Heidelberg (Bergheimer Str. 69, 69115 Heidelberg, Germany). All experiments were in accordance with the institutional guidelines (designated veterinarian according to article 25 of Directive 2010/63/EU and Animal-Welfare Body according to article 27 of Directive 2010/63/EU) and were officially approved by Regional Council of Karlsruhe (File No 35–9185.81/G-64/13 and 35–9185.81/G-200/15).

### Plasmid construction

To generate the Luc and the Luc-Cre vectors, the pS/MARt-GFP DNA vector was first digested with the restriction enzymes NheI and BglII to linearize the vector and eliminate the transgene GFP. The InFusion system provided by Clonetech was used to introduce the luciferase gene alone or in combination with the Cre recombinase gene to generate the vector pS/MARt-Luc or the vector pS/MARt-Luc-P2A-Cre, respectively.

### UVB treatments

UVB irradiation was performed with a Bio-Spectra system (Vilber Lourmat, Marne La Vallee, France) at a wavelength of 312 nm as previously described [15]. Briefly, animals were anesthetized with 3% Sevorane (Abbott, Wiesbaden, Germany) in an inhalation anesthetizer (Provet, Lyssach, Switzerland) and placed in a covered compartment with an upper square opening (3×2 cm) at a distance of 40 cm from the UVB lamp.

To study UV-induced carcinogenesis, 7-week-old female FVB/N WT or K14 HPV38 E6/E7 Tg animals were shaved on the dorsal skin with electric clippers and irradiated 3 times a week for 10 weeks with increasing doses of UVB, starting from 120 mJ/cm^2^ to a final dose of 450 mJ/cm^2^, with a constant weekly increase to allow skin thickening. For the following 20 weeks, mice were irradiated 3 times a week with 450 mJ/cm^2^. The UV irradiation protocol was based on the data described in [40] and to mimic the situation in humans. For instance, the maximum dose of the UV irradiation protocol, 450 mJ/cm^2^, corresponds to 50 minutes of solar exposure in July in Paris. The tumour incidence (tumour bearers/group) was recorded weekly. Tumours were identified first macroscopically and by histological diagnosis. After 30 weeks, or earlier if the tumour reached the ethically allowed maximal size, the animals were sacrificed and H&E-stained sections of dorsal skin were used for histological diagnosis.

### Excision of floxed viral transgenes

To study the effect of the loss of the viral genes on skin cancer development, 7-week-old K14 HPV38 E6/E7 Tg mice (*n* = 14) were shaved on the dorsal skin and treated for 30 weeks with increasing doses of UVB as previously described [15]. As soon as skin lesions (maximum diameter 2.6 mm) became evident, 46 μg of pS/MARt-Luc or 50 μg of pS/MARt-Luc-P2A-Cre dissolved in isotonic saline solution was injected directly into the lesions. To facilitate the uptake of the injected DNA, an electric field was applied to the area of the injection site using a Tweezertrodes connected to a BTX ECM 630 generator (Harvard Apparatus, Holliston, MA, USA). A first high-voltage electric pulse (1400 V/cm, 100 μs, 2 times), to induce temporary gaps in the keratinocytes cell membrane, was followed by a low-voltage electric field (140 V/cm, 400 ms, 2 times), to facilitate the migration of the DNA into the cells. At 72 h after the DNA injection, the mice were injected intraperitoneally with 150 mg/kg of luciferin in sterile water, and the luciferase activity was then assessed using an IVIS Lumina III imaging system (Perkin Elmer, Rodgau, Germany). When possible, a single mouse received both plasmids at the same time, each on a different lesion. The UV irradiation continued until week 30, according to the protocol [15]. The lesions were then closely monitored and the animals were sacrificed in accordance with an ethical protocol to avoid animal suffering. Skin lesions were collected for histological examination and detection for HPV38 E6/E7 RNA by *in situ* hybridization.

### Total RNA isolation and reverse transcription PCR analyses

Total RNA was isolated from dorsal skin of WT (*n* = 4) or K14 HPV38 E6/E7 Tg animals (*n* = 5) as well as histologically confirmed pre-malignant (pre-m) and SCC from three independent mice. cDNA was synthesized from 1 μg of total RNA using M-MLV reverse transcriptase (Invitrogen, Darmstadt, Germany), and a mix of random hexamers were used as primers. Quantitative reverse transcription PCR (RT-qPCR) was performed in a 20 μl mixture containing 1 μl of 1:10 diluted cDNA and Mesa green quantitative PCR (qPCR) Master Mix (Eurogentec, Angers, France) with specific mouse ΔNp73α primers (5′-GCCAAAAGGGTCATCATC-3′ and 5′-TGCCAGTGAGCTTCCCGTTC-3′) or mouse GAPDH primers to amplify a housekeeping gene as internal control (5′-GTGACCCCATGAGACACCTC-3′ and 5′-GTATGTCCAGGTGGCCGAC–3′), using an Applied Biosystems 7300 machine (Applied Biosystems, Darmstadt, Germany). The fluorescence threshold value was calculated using the SDS analysis software from Applied Biosystems.

### *In situ* hybridization

Once the tumours reached the maximum ethically allowed size, the mice were killed and the lesions isolated. Half of the lesion was embedded in OCT medium and slowly cooled down to −80°C. Sense and antisense riboprobes were generated from linearized plasmid DNA containing full-length HPV38E6E7 cDNA using the Digoxigenin RNA labelling Mix from Roche. RNA-RNA *in situ* hybridization was performed as previously described[41]. In brief, serial 5 μm cryo-sections were mounted on Superfrost Plus slides (Thermo Scientific), fixed in 4% paraformaldehyde in 2× SSPE, digested with proteinase K (0.5 μg/ml), and pre-hybridized at 42°C for 2–4 h. Hybridization was performed overnight at 42°C in 50% formamide, 2× SSPE, 10% dextran sulfate, 10 mM Tris-HCl pH 7.5, 1× Denhardt’s solution, 500 μg/ml tRNA, 100 μg/ml herring sperm DNA, 0.1% SDS, and 10 μg/ml DIG-labelled riboprobe. After hybridization, slides were washed once in 50% formamide, 2× SSPE; 0.1% SDS for 30 min at 50°C, treated with RNaseA (50 μg/ml in 2× SSC, 0.1% SDS), and washed again in 50% formamide, 0.5× SSPE, 0.1% SDS for 30 min at 37°C. Hybridization signals were visualized using Biotin Tyramide (TSA Biotin System, PerkinElmer) according to the manufacturer’s protocol.

### Statistical analysis

Tumour growth values of lesions injected with the pS/MARt-Luc or pS/MARt-Luc-P2A-Cre vector were compared with the two-sample *t*-test. The statistical analysis was performed with GraphPad Prism (version 6, GraphPad Software Inc., La Jolla, CA, USA).

### Exome analysis

The quality of the raw reads was estimated with FastQC software (version 0.11.5, http://www.bioinformatics.babraham.ac.uk/projects/fastqc/). Reads were mapped to the GRCm38 Mouse reference genome (ftp://hgdownload.cse.ucsc.edu/goldenPath/mm10/) using Burrows-Wheeler Aligner (BWA, http://bio-bwa.sourceforge.net/) version 0.7.15 and producing a BAM file. The following GATK Best Practice Recommendations were applied to the BAM files to improve variant detection quality. Picard (version 2.4.1, https://broadinstitute.github.io/picard/) SortSAM was used to sort and index BAM files, and the AddOrReplaceReadGroups tool was used to replace all read groups with a single new read group. The duplicate reads were marked with the MarkDuplicates tool from Picard, and the newly produced BAM file was indexed with the BuildBamIndex tool. GATK (version 3.6.0, https://software.broadinstitute.org/gatk/download/) RealignerTargetCreator was used to determine the position concerned by local realignment, and IndelRealigner was used to perform local realignment around these sites. The GATK BaseRecalibrator tool was used to detect systematic errors in base quality scores. Dbsnp and dbindel (version 142) for the mm10 reference genome was downloaded from the Sanger website (ftp://ftp-mouse.sanger.ac.uk/REL-1505-SNPs_Indels/) and considered as input. Lastly, the index of the output BAM file was created with Picard BuildBamIndex, and GATK PrintReads was used to write out sequence read data.

The quality of the alignment was estimated with Qualimap (version 2.0.2, http://qualimap.bioinfo.cipf.es/). Then the variant calling was done with Mutect (version 1.1.7, http://archive.broadinstitute.org/cancer/cga/mutect), by using a skin sample from a WT mouse not exposed to UV as the “normal sample” for paired analysis. Only somatic mutations passing Mutect internal filters were considered for the analysis. The VCF files are annotated with Annovar by using the MutSpec Annot Tool in Galaxy [42]. Variants were then filtered based on SegDup databases from UCSC (version from 4 May 2014, http://hgdownload.cse.ucsc.edu/goldenPath/mm10/database/genomicSuperDups.txt.gz), as well as Tandem Repeat and Repeat Masker (version from 9 February 2012, http://hgdownload.soe.ucsc.edu/goldenPath/mm10/bigZips/). House-made scripts were then used to keep only SNPs that have a functional impact and fall in exonic or splicing regions. Non-negative matrix factorization mutational signatures were inferred with MutSpec-NMF tools, as previously reported.

The pathway analysis was performed using the EnrichR web application (http://amp.pharm.mssm.edu/Enrichr/; citations*2). The input gene list was made by merging the mutations detected in the pre-malignant lesions (*n* = 3) or cSCCs (*n* = 3) of the K14 HPV38 E6/E7 Tg animals. The analysis included only genes harbouring mutations that are likely to alter the biological properties of the encoded products, i.e., 3111 genes in the pre-malignant lesions and 6372 genes in the cSCCs. The gene lists were then loaded into the EnrichR software, and the result from the KEGG database (version 2016) was considered. Only pathways with a significant adjusted *p*-value are shown in S1 Table. The list of pathways is ranked by combined score (combined score is computed by taking the log of the *p*-value from the Fisher exact test and multiplying it by the *z*-score of the deviation from the expected rank).

### Comparison with epigenetic driver/modifier genes and Cancer Gene Census list

The list of epigenetic driver and modifier genes was constructed on the basis of genes reported in different publications [19–23]. The Cancer Gene Census list was downloaded from the COSMIC website (12 November 2016, http://cancer.sanger.ac.uk/census) and is based on a previous publication [24].

The comparison of the mouse data with the human data [25,26] was done with Bioconductor (release 3.4, https://www.bioconductor.org/) in R (version 3.3.2, “Sincere Pumpkin Patch”). The module BioMart[43,44], version 2.3 enables the conversion of nearly 87.86% of human gene names from the Chitsazzadeh et al. publication [26] to their corresponding mouse gene names.

## Supporting information

S1 Fig Representative images of H&E-stained sections from WT or Tg mice from which the genomic DNA was extracted for exome sequencing. (A, B) Normal skin from WT (A) and K14 HPV38 E6/E7 Tg (B) mice UV-irradiated for 30 and 28 weeks, respectively. Both specimens show a clearly intact epithelium composed of a few layers of keratinocytes. (C, D) Pre-cancerous lesions from K14 HPV38 E6/E7 Tg mice UV-irradiated for 26 (C) and 28 (D) weeks, respectively. In both lesions, the keratinocytes present acanthosis, diffused intraepithelial atypia, and a high number of mitosis; an intact basal membrane is evident. Enlargements of the most affected areas are displayed. (E, F). Cancerous lesions (SCC) from K14 HPV38 E6/E7 Tg mice UV-irradiated for 26 (E) and 28 (F) weeks, respectively. Both sections are characterized by the presence of polymorphic tumour cells with big nuclei, diffused presence of horn pearls, and hyperkeratinization. The enlargements show tumour invasion of the subcutaneous fat (E) or of muscle fibres (F). The stained sections were first scanned with no enlargement and then zoomed in via software analysis.
(TIF)

S2 Fig Mutational signature detected in skin keratinocytes of UV-irradiated K14 HPV38 E6/E7 Tg mice. (A) Mutational signature obtained after applying the NMF method to all 9 samples (3 normal skin, 3 pre-malignant lesions, and 3 SCCs). (B) The B signature shows a strong identity with the UV signature (cosine similarity of 0.86). (C) The SCC and pre-malignant samples of the different mice are the main contributors to inference of the B signature.
(TIF)

S3 Fig Modulation of HPV38 E6 and E7 expression in skin keratinocytes of K14 Cre-ERT2 HPV38 E6/E7 Tg mice. Total RNA was extracted from dorsal skin keratinocytes from K14 HPV38 E6/E7 Tg mice (10-week-old, *n* = 3; 30-week-old, *n* = 3), and from Cre-ERT2 HPV38 E6/E7 Tg mice, treated (10-week-old, *n* = 9; 30-week-old, *n* = 4) or not (10-week-old, *n* = 7; 30-week-old, *n* = 4)) with 4-hydroxytamoxifen (TMX). HPV38 E6 mRNA quantification was performed by quantitative RT-PCR. The relative quantification + SD is shown. The following differences are statistically significant according to *t*-test analysis: 10-week-old K14 HPV38 E6/E7 Tg vs 10-week-old K14 Cre-ERT2 HPV38 E6/E7 Tg, *p* < 0.05; 10-week-old K14 HPV38 E6/E7 Tg vs 30-week-old K14 Cre-ERT2 HPV38 E6/E7 Tg, *p* < 0.0001; 10-week-old K14 HPV38 E6/E7 Tg vs 10-week-old K14 Cre-ERT2 HPV38 E6/E7 Tg + TMX, *p* < 0.0001; 10-week-old K14 HPV38 E6/E7 Tg vs 30-week-old K14 Cre-ERT2 HPV38 E6/E7 Tg + TMX, *p* < 0.0001; 10-week-old K14 Cre-ERT2 HPV38 E6/E7 Tg vs 10-week-old K14 Cre-ERT2 HPV38 E6/E7 Tg + TMX, *p* < 0.0001; 10-week-old K14 Cre-ERT2 HPV38 E6/E7 Tg vs 30-week-old K14 Cre-ERT2 HPV38 E6/E7 Tg, *p* < 0.01; 30-week-old K14 HPV38 E6/E7 Tg vs 30-week-old K14 Cre-ERT2 HPV38 E6/E7 Tg, *p* < 0.05; 30-week-old K14 Cre-ERT2 HPV38 E6/E7 Tg vs 30-week-old K14 Cre-ERT2 HPV38 E6/E7 Tg + TMX, *p* < 0.01.
(TIF)

S1 Table Mutated genes in animals without skin lesions. Cellular pathways were linked to the different gene products using the information at http://www.genecards.org.
(DOCX)

S2 Table Global view of the somatic mutations and coverage of the sequencing of skin samples from different mice (M1–3). (DOCX)

S3 Table Pathway analyses. The pathway deregulated in the pre-malignant lesions (A) or cSCC (B). The gene list used as input is the consensus of the genes mutated in the different pre-malignant samples. Only the significant pathways (adjusted *p*-value > 0.05) are shown.
(DOCX)

S4 Table Trp53 nonsynonymous mutations in the DNA-binding domain detected in normal skin, pre-malignant lesions, and cSCCs. (DOCX)
