# Supplementary figures and images for: Beta HPV38 oncoproteins act with a hit-and-run mechanism in ultraviolet radiation-induced skin carcinogenesis in mice
Source: PLoS Pathog. 2018 Jan 11;14(1):e1006783. doi: 10.1371/journal.ppat.1006783 (PMC5764406; doi:10.1371/journal.ppat.1006783)

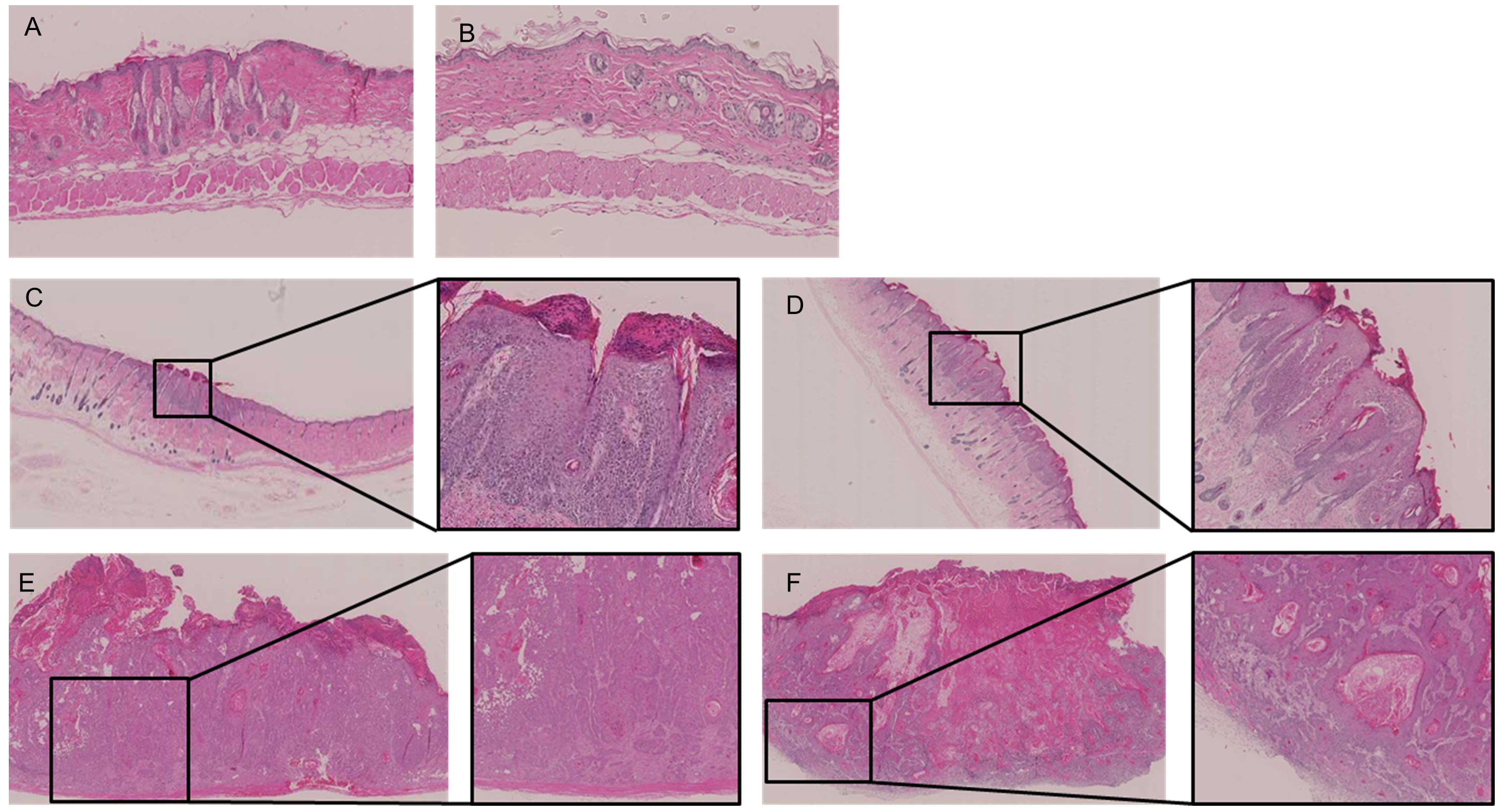

Supplement: S1 Fig — (A, B) Normal skin from WT (A) and K14 HPV38 E6/E7 Tg (B) mice UV-irradiated for 30 and 28 weeks, respectively. Both specimens show a clearly intact epithelium composed of a few layers of keratinocytes. (C, D) Pre-cancerous lesions from K14 HPV38 E6/E7 Tg mice UV-irradiated for 26 (C) and 28 (D) weeks, respectively. In both lesions, the keratinocytes present acanthosis, diffused intraepithelial atypia, and a high number of mitosis; an intact basal membrane is evident. Enlargements of the most affected areas are displayed. (E, F). Cancerous lesions (SCC) from K14 HPV38 E6/E7 Tg mice UV-irradiated for 26 (E) and 28 (F) weeks, respectively. Both sections are characterized by the presence of polymorphic tumour cells with big nuclei, diffused presence of horn pearls, and hyperkeratinization. The enlargements show tumour invasion of the subcutaneous fat (E) or of muscle fibres (F). The stained sections were first scanned with no enlargement and then zoomed in via software analysis. (TIF) [file ppat.1006783.s001.tif]

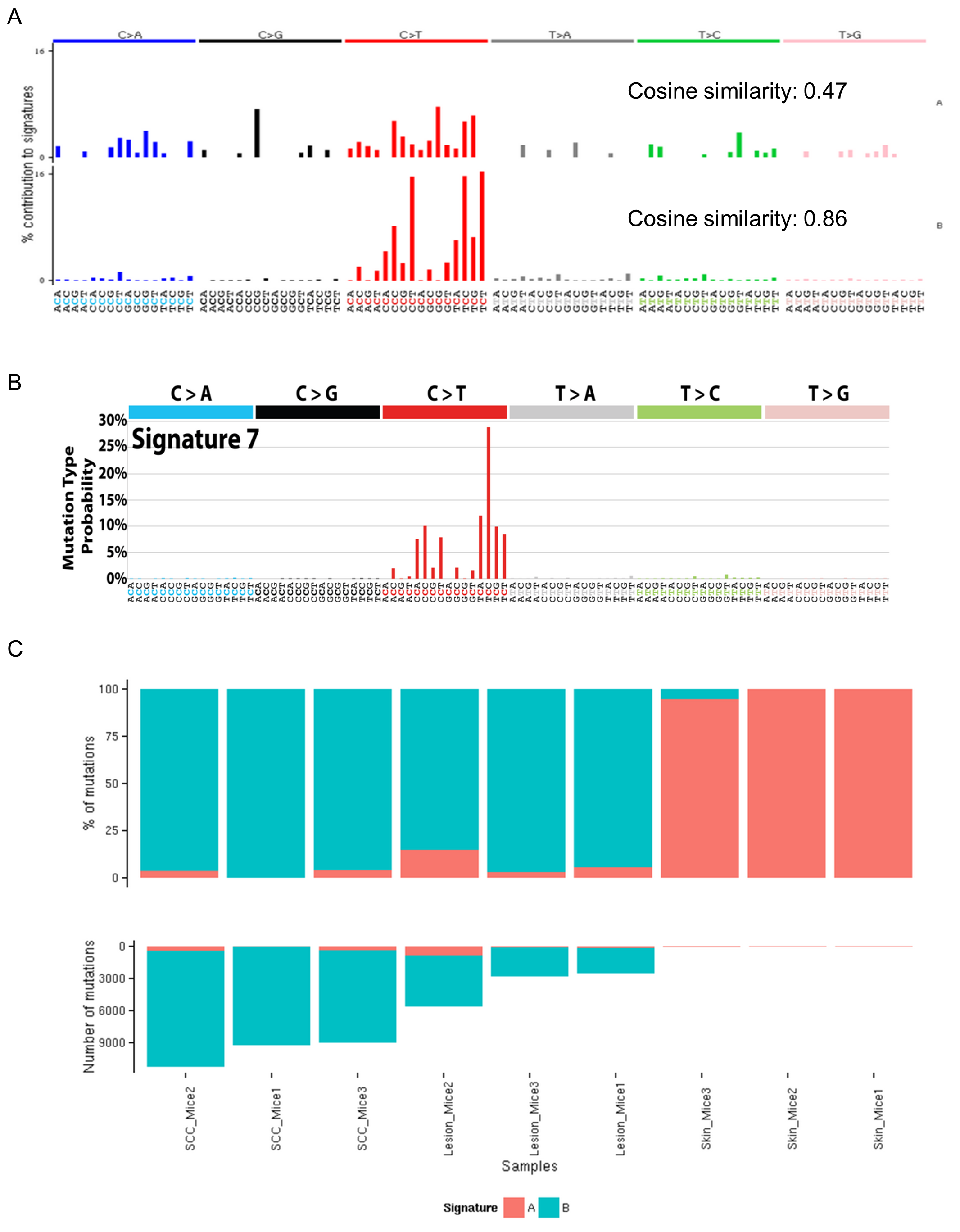

Supplement: S2 Fig — (A) Mutational signature obtained after applying the NMF method to all 9 samples (3 normal skin, 3 pre-malignant lesions, and 3 SCCs). (B) The B signature shows a strong identity with the UV signature (cosine similarity of 0.86). (C) The SCC and pre-malignant samples of the different mice are the main contributors to inference of the B signature. (TIF) [file ppat.1006783.s002.tif]

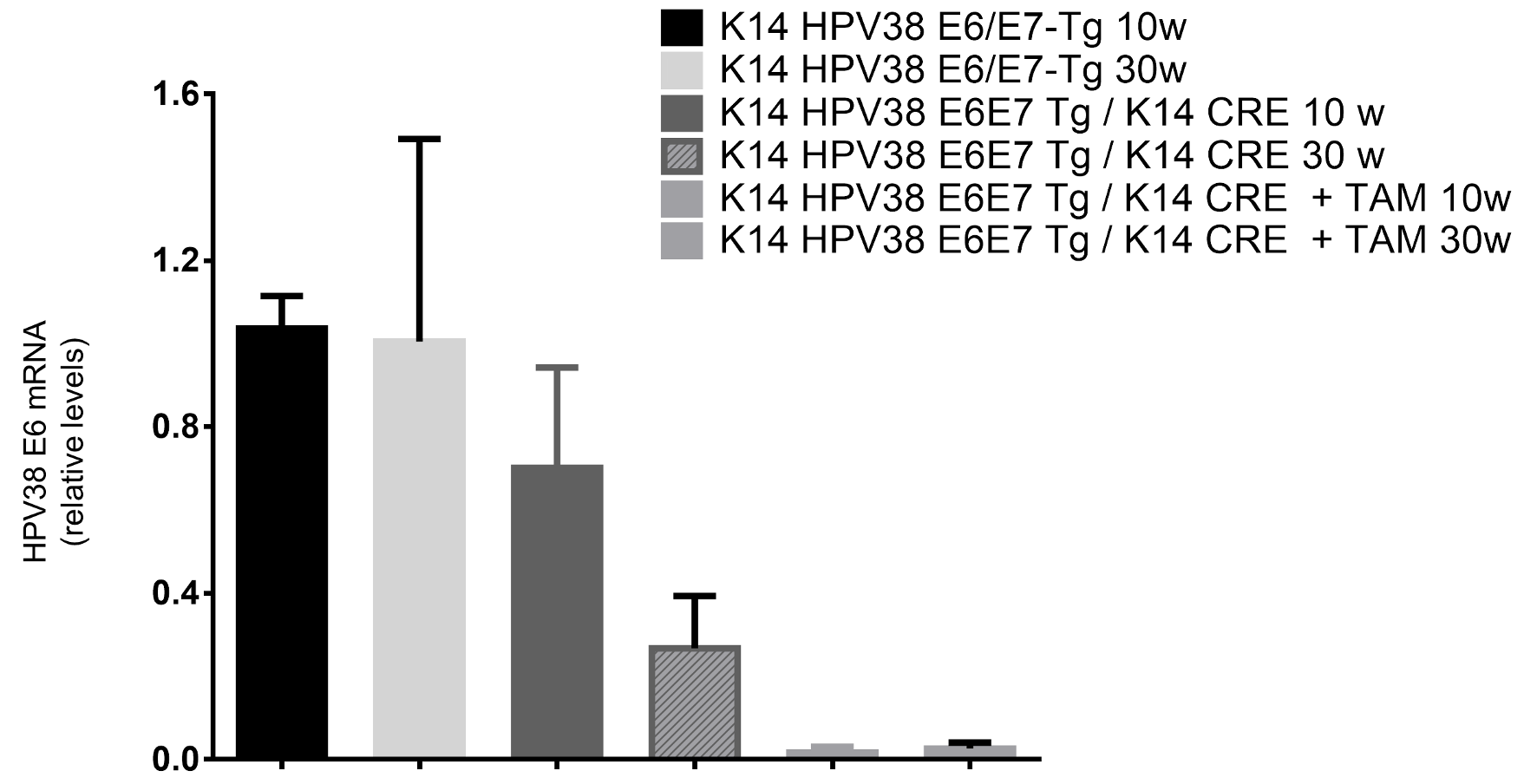

Supplement: S3 Fig — Total RNA was extracted from dorsal skin keratinocytes from K14 HPV38 E6/E7 Tg mice (10-week-old, n = 3; 30-week-old, n = 3), and from Cre-ERT2 HPV38 E6/E7 Tg mice, treated (10-week-old, n = 9; 30-week-old, n = 4) or not (10-week-old, n = 7; 30-week-old, n = 4)) with 4-hydroxytamoxifen (TMX). HPV38 E6 mRNA quantification was performed by quantitative RT-PCR. The relative quantification + SD is shown. The following differences are statistically significant according to t-test analysis: 10-week-old K14 HPV38 E6/E7 Tg vs 10-week-old K14 Cre-ERT2 HPV38 E6/E7 Tg, p < 0.05; 10-week-old K14 HPV38 E6/E7 Tg vs 30-week-old K14 Cre-ERT2 HPV38 E6/E7 Tg, p < 0.0001; 10-week-old K14 HPV38 E6/E7 Tg vs 10-week-old K14 Cre-ERT2 HPV38 E6/E7 Tg + TMX, p < 0.0001; 10-week-old K14 HPV38 E6/E7 Tg vs 30-week-old K14 Cre-ERT2 HPV38 E6/E7 Tg + TMX, p < 0.0001; 10-week-old K14 Cre-ERT2 HPV38 E6/E7 Tg vs 10-week-old K14 Cre-ERT2 HPV38 E6/E7 Tg + TMX, p < 0.0001; 10-week-old K14 Cre-ERT2 HPV38 E6/E7 Tg vs 30-week-old K14 Cre-ERT2 HPV38 E6/E7 Tg, p < 0.01; 30-week-old K14 HPV38 E6/E7 Tg vs 30-week-old K14 Cre-ERT2 HPV38 E6/E7 Tg, p < 0.05; 30-week-old K14 Cre-ERT2 HPV38 E6/E7 Tg vs 30-week-old K14 Cre-ERT2 HPV38 E6/E7 Tg + TMX, p < 0.01. (TIF) [file ppat.1006783.s003.tif]

Table S3A


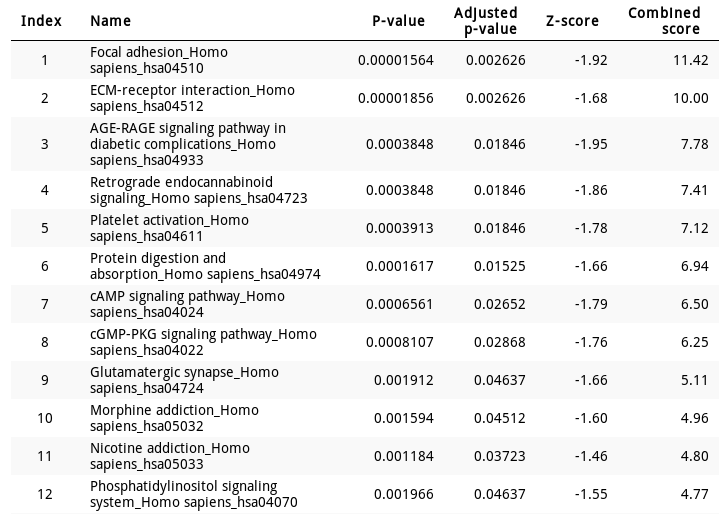


Table S3B


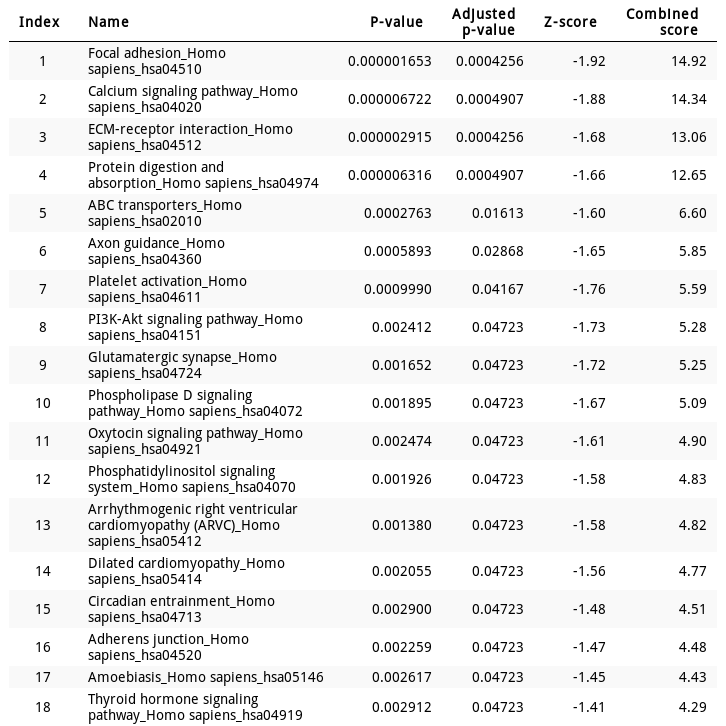

Supplement: S3 Table — The pathway deregulated in the pre-malignant lesions (A) or cSCC (B). The gene list used as input is the consensus of the genes mutated in the different pre-malignant samples. Only the significant pathways (adjusted p-value > 0.05) are shown. (DOCX) [file ppat.1006783.s006.docx]
