# Supplementary material for: Beta HPV38 oncoproteins act with a hit-and-run mechanism in ultraviolet radiation-induced skin carcinogenesis in mice
Source: PLoS Pathog. 2018 Jan 11;14(1):e1006783. doi: 10.1371/journal.ppat.1006783 (PMC5764406; doi:10.1371/journal.ppat.1006783)
Supplement: S1 Table — Cellular pathways were linked to the different gene products using the information at http://www.genecards.org. (DOCX) [file ppat.1006783.s004.docx]

Table S1

| **WT animal exposed to 30 week UV-irradiation** | | | | | | |
| --- | --- | --- | --- | --- | --- | --- |
| **Gene name** | | **Pathway/cellular event** | **DNA mutation** | | **Amino acid change** | |
| Unc80 | | Ion channel transport | Chr1:66654398-66654398:T>G | | L2536A | |
| Akr1c14 | | Aldo-Keto Reductase Family 1 Member C14 | Chr13:4078030:4078030:C>A | | L144I | |
| Znfx1 | RNA binding/transcription factor activity, sequence-specific DNA binding | | | Chr2:167056200-167056200:G>T | | T268N |
| Taf4a | RNA Polymerase II Transcription Initiation And Promoter Clearance | | | Chr2:179931984-179931984:G>A | | P697L |
| Nup188 | Transport of the SLBP independent Mature mRNA | | | Chr2:30330652-30330652:G>T | | M912I |
| Olfr653 | Olfactory receptor activity | | | Chr7:104579982-104579982:C>T | | A112V |
| Itgal | Blood-Brain Barrier and Immune Cell Transmigration: VCAM-1/CD106 Signaling Pathways | | | Chr7:127328539-127328539:C>T | | P1002L |
| Pkd1l3 | Taste transduction | | | Chr8:109623606-109623606:T>C | | L361S |
| Sik3 | LKB1 signaling events | | | Chr9:46195266-46195266:G>C | | S399T |
| **K14 HPV38 E6/E7 Tg not exposed to UV irradiation** | | | | | | |
| **Gene name** | | **Pathway/cellular event** | **DNA mutation** | | **Amino acid change** | |
| Itln1 | | Innate Immune System | Chr1:171518245-171518245:C>T | | R331H | |
| Adamts20 | | O-glycosylation of TSR domain-containing proteins | Chr15:94327776-94327776:C>T | | G1147E | |
| Pfkm | | Glucose metabolism | Chr15:98128333-98128333:G>T | | R603L | |
| Vps52 | | Vesicle-mediated transport | Chr17:33962919-33962919:G>C | | E544Q | |
| Zfp236 | | Unknown | Chr18:82671742-82671742:C>A | | C204F | |
| Zfp69 | | Gene Expression | Chr4:120947329-120947329:T>C | | H115R | |
