# Supplementary material for: Beta HPV38 oncoproteins act with a hit-and-run mechanism in ultraviolet radiation-induced skin carcinogenesis in mice
Source: PLoS Pathog. 2018 Jan 11;14(1):e1006783. doi: 10.1371/journal.ppat.1006783 (PMC5764406; doi:10.1371/journal.ppat.1006783)
Supplement: S2 Table — (DOCX) [file ppat.1006783.s005.docx]

Table S2

| **Type of sample** | **Number of somatic mutations in total (Output Mutect PF − Impact mutations** | **Number of somatic mutations per Mb** | **Coverage (mean ± SD)** |
| --- | --- | --- | --- |
| Normal skin (M1) | 11 | 0.21 | 123.50 ± 397.84 |
| Normal skin (M2) | 16 | 0.30 | 137.32 ± 1242.80 |
| Normal skin (M3) | 20 | 0.38 | 161.78 ± 789.92 |
| Pre-malignant lesion (M1) | 937 | 10.08 | 139.00 ± 390.92 |
| Pre-malignant lesion (M2) | 2026 | 39.11 | 121.46 ± 368.06 |
| Pre-malignant lesion (M3) | 1048 | 20.23 | 130.34 ± 609.97 |
| cSCC (M1) | 3261 | 63.94 | 151.21 ± 1187.73 |
| cSCC (M2) | 4027 | 77.40 | 152.89 ± 753.97 |
| cSCC (M3) | 3336 | 64.94 | 157.95 ± 880.89 |
