# Supplementary material for: Beta HPV38 oncoproteins act with a hit-and-run mechanism in ultraviolet radiation-induced skin carcinogenesis in mice
Source: PLoS Pathog. 2018 Jan 11;14(1):e1006783. doi: 10.1371/journal.ppat.1006783 (PMC5764406; doi:10.1371/journal.ppat.1006783)
Supplement: S4 Table — (DOCX) [file ppat.1006783.s007.docx]

Table S4

| **Type of specimens** | **Nucleotide change** | **Amino acid change** |
| --- | --- | --- |
| Normal skin | C628T | R210C |
|  | C808T | R270C |
| Pre-malignant lesions | C371T | S124F |
|  | C379T | L127F |
|  | C442T | P148S |
|  | C628T | R210C |
|  | C808T | R270C |
| cSCCs | C371T | S124F |
|  | C571T | L191F |
|  | C628T | R210C |
|  | C808T | R270C |
|  | C824T | P275L |
